# The Annual Economic Burden of Respiratory Syncytial Virus in Adults in the United States

**DOI:** 10.1093/infdis/jiad559

**Published:** 2023-12-07

**Authors:** Justin Carrico, Katherine A Hicks, Eleanor Wilson, Catherine A Panozzo, Parinaz Ghaswalla

**Affiliations:** RTI Health Solutions, Durham, North Carolina, USA; RTI Health Solutions, Durham, North Carolina, USA; Moderna, Inc, Cambridge, Massachusetts, USA; Moderna, Inc, Cambridge, Massachusetts, USA; Moderna, Inc, Cambridge, Massachusetts, USA

**Keywords:** burden of disease, cost of illness, respiratory syncytial virus, adults, high-risk adults

## Abstract

**Background:**

Current estimates of the economic burden of respiratory syncytial virus (RSV) are needed for policymakers to evaluate adult RSV vaccination strategies.

**Methods:**

A cost-of-illness model was developed to estimate the annual societal burden of RSV in US adults aged ≥60 years. Additional analyses were conducted to estimate the burden of hospitalized RSV in all adults aged 50–59 years and in adults aged 18–49 years with potential RSV risk factors.

**Results:**

Among US adults aged ≥60 years, the model estimated 4.0 million annual RSV cases (95% uncertainty interval [UI], 2.7–5.6 million) and an annual economic burden of $6.6 billion (95% UI, $3.1–$12.9 billion; direct medical costs, $2.9 billion; indirect costs, $3.7 billion). The 4% of RSV cases that were hospitalized contributed to 94% of direct medical costs. Additional analyses estimated $422 million in annual hospitalization costs among all adults aged 50–59 years. Among adults aged 18–49 years with RSV risk factors, annual per capita burden was highest among people with congestive heart failure at $51 100 per 1000 people.

**Discussion:**

The economic burden of RSV is substantial among adults aged ≥50 years and among adults aged 18–49 years with RSV risk factors, underscoring the need for preventive interventions for these populations.

## Teaser

RSV cases in US adults aged ≥60 years were estimated to contribute a total annual cost of $6.6 billion (95% uncertainty interval, $3.1–$12.9 billion), including $2.9 billion in direct costs and $3.7 billion in indirect costs.

Respiratory syncytial virus (RSV) is a common and highly infectious respiratory virus that can cause severe disease in older adults and adults with underlying comorbidities (eg, chronic lung and heart disease), leading to an exacerbation of chronic illness, hospitalization, and in some cases, death [1–4]. A recent meta-analysis estimated that 5.2 million cases of RSV-associated acute respiratory infection occur in high-income countries annually, with approximately 302 000 to 720 000 hospitalizations and 16 000 to 67 000 deaths during hospitalizations among adults aged ≥60 years [5]. A subset of older adults hospitalized for RSV require a higher level of care after hospitalization [6]. As the global population ages, the morbidity and mortality associated with respiratory infections such as RSV are expected to steadily increase. While the risk factors for severe RSV are less studied relative to other common respiratory viruses, available evidence suggests that adults of all ages with chronic obstructive pulmonary disease (COPD), congestive heart failure (CHF), coronary artery disease (CAD), asthma, or diabetes may be at increased risk of developing RSV that results in hospitalization [1–4].

Although the economic burden of other common respiratory infections, such as influenza, is well studied in adults [7–11], fewer estimates of the RSV burden in adults are available. A recent modeling study estimated an annual RSV burden of nearly $3 billion (2019 US dollars) from a health care payer perspective for older adults in the United States [12]. To our knowledge, the societal economic burden, which includes both direct and indirect costs of RSV, has not been estimated for US adults. Additionally, the economic burden of RSV in high-risk younger US populations is not well quantified, and these younger groups tend to have higher indirect costs due to lost time from paid employment, as previously estimated for influenza infections [8]. Furthermore, indirect costs are generally not sufficiently recognized by decision makers in economic evaluations of vaccination programs [13].

An RSV vaccine for adults, particularly older adults, could help prevent primary infection and help preserve independence, health, and quality of life. Until recently, there were no RSV vaccines approved for use in any country. However, in May 2023, the United States Food and Drug Administration and the European Medicines Agency approved 2 vaccines for the prevention of RSV in adults aged ≥60 years [14–17]. A recommendation for a single dose of RSV vaccine in adults aged ≥60 years based on shared clinical decision-making was subsequently issued by the Advisory Committee on Immunization Practices [18]. Cost-of-illness modeling studies can estimate the health and economic impact of RSV in various populations and potentially inform updates to recommendations focused on mitigating the RSV impact through immunization practices.

The objective of this analysis was to estimate the economic burden of RSV in adults recognized as high risk for severe RSV, specifically the total economic burden in all adults aged ≥60 years, hospitalized burden in all adults aged 50 to 59 years, and hospitalized burden in adults aged 18 to 49 years with potential RSV risk factors.

## METHODS

### Model and Population Overview

**Figure 1.**
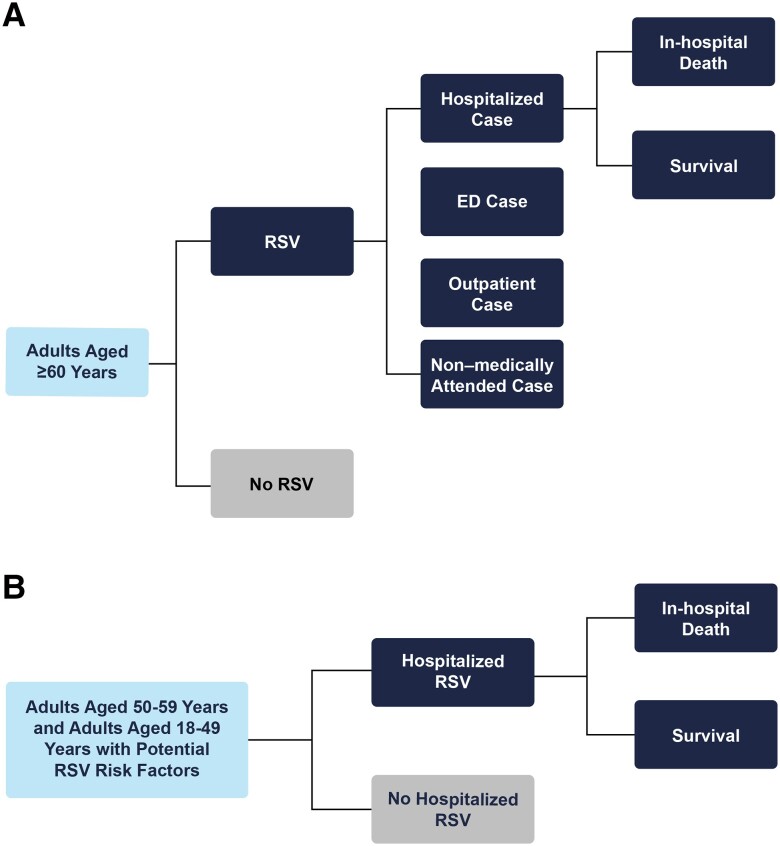
Model diagram. *A*, The number of RSV cases treated in outpatient, ED, and hospital settings were calculated from data on the distribution of health care resource use from RSV cases; the remaining cases were assumed to not be medically attended. RSV-related deaths occurring during hospitalized cases were also accounted for. *B*, The model's decision tree structure for additional analyses of the burden of hospitalized RSV in adults aged 50–59 years and adults aged 18–49 years with potential RSV risk factors. Nonhospitalized RSV cases were not considered in these age groups due to limited data availability. Abbreviations: ED, emergency department; RSV, respiratory syncytial virus.

This study used a cost-of-illness model to estimate clinical outcomes and economic costs associated with RSV infection. Clinical outcomes included RSV-associated cases, hospitalizations, emergency department and outpatient visits, and RSV-related deaths occurring during hospitalized cases in the United States over a 1-year time period (Figure 1*A*). The model considered a societal economic perspective, including direct medical costs from RSV-associated health care resource utilization, as well as indirect costs for productivity losses from RSV-associated morbidity and, optionally, deaths. All economic costs were reported in 2021 US dollars. The base-case analysis population was defined as US adults aged ≥60 years (older adults); additional analyses were also conducted in the population aged 50 to 59 years and in adults aged 18 to 49 years with potential RSV risk factors (Figure 1*B*). Of note, for the calculation of the economic burden of RSV in older adults, the population was also grouped into 2 categories: those at high risk of severe disease from RSV and those not at high risk of severe disease. Per the Centers for Disease Control and Prevention (CDC), high-risk conditions for severe disease in older adults included chronic lung or heart disease, including COPD, CHF, CAD, or asthma [1, 3, 4].

### Epidemiology Inputs

Detailed information on the epidemiologic input values for both base-case and scenario analyses are shown in Table 1. The annual incidence of RSV and associated health care resource utilization rates were obtained from a landmark prospective study of adults in Rochester, New York [1]; the study reported RSV incidence for cohorts of adults with chronic heart or lung disease (high risk) and adults with no disabling underlying illnesses (non–high risk). Overall incidence in adults aged ≥60 years was calculated as a weighted average of the incidence in the high-risk and non–high-risk groups using weights reflecting the percentage of the US population with COPD, CHF, or CAD, as estimated from 2017–2018 National Health and Nutrition Examination Survey data [20]. RSV-related mortality was assumed to occur during 4% to 6% of hospitalizations among older adults from 2015–2019 RSV-NET data [21] and 2% to 3% of hospitalizations among adults aged 18 to 59 years from 2022–2023 RSV-NET data [22].

**Table 1.** Base-Case and Additional Analyses Inputs

Parameter	Value (Range^a^)
US population size by age group, n [19]	
18–49 y	140 148 894
50–59 y	41 774 561
60–64 y	21 118 423
65–69 y	18 631 422
70–79 y	26 018 017
≥80 y	13 145 413
Population aged ≥60 y at high risk for severe RSV infection, %^b^ [20]	
60–69 y	18.2 (14.5–21.8)
70–79 y	22.5 (18.0–27.0)
≥80 y	29.7 (23.7–35.6)
Annual incidence of RSV per 1000 person-years in adults aged ≥60 y^c^ [1, 20]	
Non–high risk	47.6 (27.8–71.4)
High risk	63.5 (35.9–97.1)
Location-of-care distribution of RSV cases in adults aged ≥60 y, % of cases^d^ [1, 20]	
Non–high risk	
Outpatient	17.4 (13.9–20.9)
Non–medically attended	82.6
High risk	
Hospitalized	16.1 (12.9–19.3)
ED	8.9 (7.1–10.7)
Outpatient	28.6 (22.9–34.3)
Non–medically attended	46.4
Case-fatality rate during RSV hospitalization, % of hospitalized cases^b^ [21, 22]	
18–49 y	2.0
50–59 y	3.0
60–64 y	3.9 (3.1–4.7)
65–74 y	4.3 (3.4–5.2)
≥75 y	5.7 (4.6–6.8)
Direct medical cost per hospitalized RSV case^e,f^ [20, 23–25]	
18–49 y	$17 634
50–59 y	$16 811
60–69 y	$15 901 ($8854–$18 746)
70–79 y	$15 458 ($8854–$18 746)
≥80 y	$14 727 ($8854–$18 746)
Direct medical cost per ED RSV case^b,e^ [26]	
18–44 y	$549
45–64 y	$706 ($564–$847)
≥65 y	$773 ($618–$927)
Direct medical cost per outpatient RSV case^b,e^ [27]	$130 ($104–$156)
Indirect cost per day of productivity loss^b,e^ [28]	
15–24 y	$66
25–34 y [29]	$211
35–44 y	$284
45–54 y	$272
55–64 y	$222 ($178–$266)
65–74 y	$126 ($101–$151)
75–99 y	$52 ($42–$63)
Days of productivity loss per RSV case	
Hospitalized^g^ [1, 3]	17.0 (7.5–20.4)
ED^h^ [1, 29]	3.5 (2.5–4.5)
Outpatient^h^ [1, 29]	2.5 (1.5–3.5)
Non–medically attended^i^ [7]	
18–49 y	0.5
50–64 y	0.5 (0–1.0)
≥65 y	1.0 (0.5–1.5)
Lifetime productivity loss due to RSV mortality^j^	
20 y	$2 284 491
40 y	$1 979 346
60 y	$782 026
80 y	$124 917
Additional analyses: burden of hospitalized RSV in all adults aged 50–59 y	
Annual incidence of hospitalized RSV per 100 000 person-years [3]	49.7
Additional analyses: burden of hospitalized RSV in adults aged 18–49 y with potential RSV risk factors	
US population with risk factor, 18–49 y, % [20]	
COPD	0.7
Asthma	16.0
Diabetes	4.2
Obesity	41.6
CAD	0.4
CHF	0.5
Annual incidence of hospitalized RSV, per 100 000 person-years [3]	
COPD	34.7
Asthma	15.2
Diabetes	75.3
Obesity	9.6
CAD	31.6
CHF	201.0–352.9

Costs are presented in 2022 US dollars.

Abbreviations: CAD, coronary artery disease; CHF, congestive heart failure; COPD, chronic obstructive pulmonary disease; ED, emergency department; RSV, respiratory syncytial virus.

^a^Ranges are only presented for inputs in adults aged ≥60 years, as sensitivity analyses were conducted for the estimated burden in older adults only.

^b^Assumed ± 20% variation from base-case values for the range.

^c^Estimates for healthy and high-risk populations presented in Falsey et al [1] were weighted by age-specific percentage of the population with COPD, CHF, or CAD in the United States [20]. The lower and upper bound incidence values reflected the low-incidence and high-incidence seasons observed in Falsey et al [1], respectively. Estimates presented in Falsey et al [1] for adults aged ≥65 years were also applied to adults aged 60–64 years in this study.

^d^Assumed ± 20% variation from base-case values for the range. For the location-of-care distribution of RSV cases, the percentage of cases that are non–medically attended was adjusted to absorb the change in the lower/upper bound value for the percentage of cases that are hospitalized, ED, or outpatient.

^e^Total annual direct and indirect costs were calculated using the number of RSV cases with each outcome and the health care resource use associated with those RSV outcomes. Indirect costs related to morbidity associated with RSV cases are calculated by multiplying age-specific daily productivity by number of days of productivity loss associated with each RSV case outcome. Values are based on average costs.

^f^Lower bound and upper bound values obtained from Choi et al [25] and Ackerson et al [24], respectively.

^g^Lower bound obtained from Branche et al [3]. Upper bound assumed +20% variation from base-case value for the range.

^h^Assumed ± 1-day variation from base-case values for the range.

^i^Assumed ± 0.5-day variation from base-case values for the range.

^j^Lifetime productivity losses varied by individual age; for simplicity, lifetime productivity losses for selected ages only are displayed in the table.

### Cost Inputs

Direct medical costs of RSV cases included costs of health care resource utilization of RSV infection treated in the hospital, emergency department, and outpatient care settings. Costs were obtained from the published literature and national unit reimbursement rates for outpatient services (Table 1). Costs of RSV hospitalizations were calculated by subtracting inpatient costs before RSV diagnosis from inpatient costs after RSV diagnosis for RSV cases among a population of Medicare beneficiaries aged ≥18 years [23]. Cost estimates from the study, which included cohorts of high-risk adults (ie, adults with chronic lung disease, prior pneumonia, CHF, or compromised immunity) and non–high-risk adults, were weighted by the percentage of the US population with COPD, CHF, or CAD [20]. Direct medical costs from RSV cases treated in an emergency department setting were obtained from a 2017 analysis of Healthcare Costs and Utilization Project data [26]. The 2022 cost of one 30- to 39-minute physician office visit for an established patient was assumed for RSV cases treated in an outpatient setting and was identified through the Medicare Physician Fee Schedule look-up tool that provides pricing information by procedure code (Current Procedural Terminology code 99214) [27]. Direct medical costs were adjusted to 2022 US dollars using the health care component of the Personal Consumption Expenditures index [30].

Indirect costs in the base-case analysis included work and nonwork (including household activities, caring, and helping people, consumer purchases, volunteer activities, secondary childcare, and other secondary eldercare) productivity losses associated with morbidity and mortality due to RSV cases [28]. These productivity data were used to derive daily productivity estimates, which were then multiplied by days of productivity loss due to medically attended and non–medically attended RSV cases. Days of lost productivity for hospitalized RSV cases were equal to the hospitalization mean length of stay (14 days) [1], plus an assumed additional 3 days of recovery posthospitalization [8]. Data for lost productivity due to RSV were limited for nonhospitalized cases, so estimates for influenza were used when data were unavailable [1, 7, 29]. Forty-three percent of outpatient RSV cases were estimated to incur lost productivity [1], with a 6-day duration of productivity losses assumed [29]. RSV cases treated in an emergency department setting were assumed to incur an additional day of productivity loss relative to outpatient RSV cases to reflect increased severity of illness. Non–medically attended RSV cases were assumed to incur the same lost productivity as non–medically attended influenza cases [7].

Productivity losses associated with deaths during RSV hospitalizations were calculated as the product of lifetime productivity loss due to death by age and the number of deaths by age during RSV hospitalizations estimated by the model. Lifetime productivity loss was estimated from remaining lifetime market and nonmarket productivity estimates at the age of death, with an assumed 0.5% annual productivity growth and a 3% discount rate [28]. Indirect costs were inflated to 2022 US dollars using the employment cost index [31].

### One-Way Sensitivity Analyses

One-way sensitivity analyses were conducted to estimate the sensitivity of total societal economic burden estimates in older adults when varying the model's inputs or groups of related inputs, one at a time. Model inputs were varied using 95% confidence intervals (CIs) or other variance data (Table 1). Where CIs or other statistical uncertainty data were not available, a range of ±20% from the base-case value(s) was used. The influence of each input was measured by the difference between the total societal costs observed when using the upper value(s) and the lower value(s) of the input's range.

### Probabilistic Sensitivity Analyses

Probabilistic sensitivity analyses were performed to assess the uncertainty around the estimates of total economic burden, annual RSV cases, and hospitalized cases in older adults, given the collective uncertainty around the model's input values. Prevalence, incidence, and case-fatality rate were sampled across β distributions, cost parameters across γ distributions, and location-of-care distribution parameters across Dirichlet distributions [32]. β distribution was used for epidemiologic inputs in accordance with the standard statistical methodology for binomial data [32]. The selection of γ distribution for cost parameters was guided by standard statistical methodology for right-skew parameters [32]. The distribution parameters were determined to be consistent with the uncertainty ranges around the inputs (Table 1), representing 95% CIs. Five thousand Monte Carlo simulations of the model were conducted, with mean values and 95% uncertainty intervals (UIs) presented for RSV cases, hospitalizations, and societal costs.

### Additional Analyses

Limited epidemiologic data were available to estimate the burden of nonhospitalized RSV adults aged <60 years. Thus, 2 additional analyses were conducted to estimate the societal economic burden of hospitalized RSV in adults aged 50 to 59 years and in adults aged 18 to 49 years with potential RSV risk factors (COPD, CHF, CAD, asthma, diabetes, and obesity [3]). The prevalence of potential RSV risk factors in adults aged 18 to 49 years was estimated from the 2017–2018 National Health and Nutrition Examination Survey data (Table 1) [20]. The annual incidence of hospitalized RSV in each of these additional analyses was obtained from a prospective population-based surveillance study conducted in New York between 2017 and 2020 (Table 1) [3].

## RESULTS

### Base-Case Analyses

**Figure 2.**
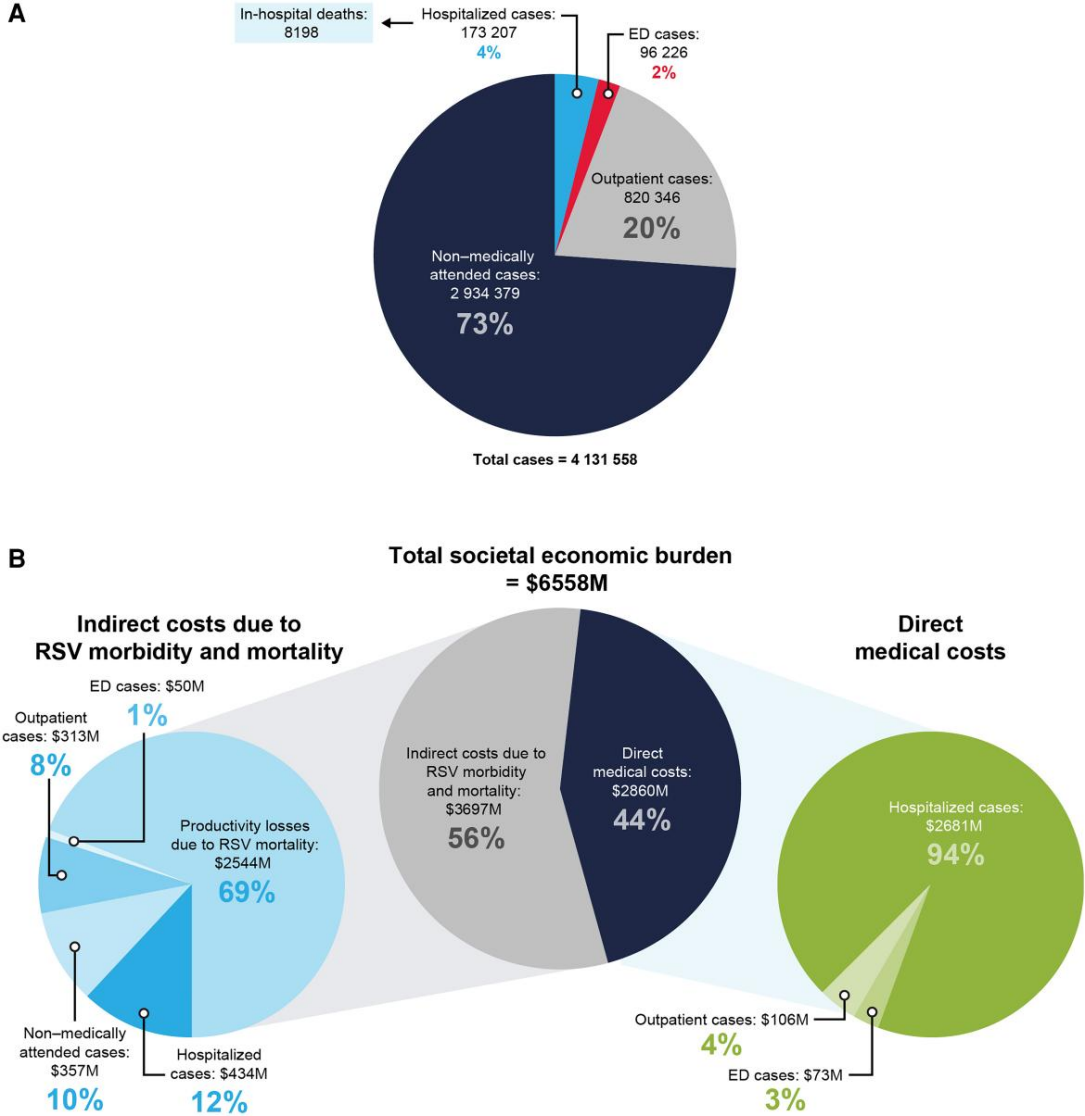
Estimates of health and economic burden of RSV in US adults aged ≥60 years. *A*, morbidity and mortality. *B*, Societal economic burden, inclusive of direct medical costs and indirect medical costs. Abbreviations: ED, emergency department; M, million; RSV, respiratory syncytial virus.

Among US adults aged ≥60 years, the model estimated an annual 4.0 million RSV infections (Figure 2*A*). These infections resulted in 1 090 000 medically attended cases (27.1%), including 820 000 outpatient cases (20.4%), 96 000 emergency department visits (2.4%), 173 000 hospitalizations (4.3%) (Figure 2*A*), and 8200 deaths during RSV-related hospitalizations. Deaths during RSV hospitalizations were numerically highest in those aged 70–79 years (2900) (Table 2).

**Table 2.** Base-Case Analysis Results: Annual Burden of RSV in Adults Aged 60 Years and Older by Age Group

Outcome	Age Group, y				
	≥60	60–64	65–69	70–79	≥80
Total annual RSV cases	4 024 158	1 065 670	940 172	1 330 896	687 421
Total annual RSV hospitalizations	173 207	39 138	34 529	59 744	39 796
Total annual deaths during RSV hospitalizations	8198	1526	1485	2918	2268
Total annual economic burden, $M	6558	2066	1482	2038	971
Direct medical costs, $M	2860	665	588	985	622
Indirect costs due to RSV morbidity and mortality, $M	3697	1401	894	1054	348
Total annual economic burden per 1000 people, $	83 100	97 833	79 568	78 346	73 846
Total direct cost burden per 1000 people, $	36 247	31 501	31 571	37 843	47 336
Total indirect cost burden per 1000 people, $	46 854	66 332	47 998	40 503	26 509

Abbreviations: M, million; RSV, respiratory syncytial virus.

RSV cases were estimated to contribute to a total annual economic burden of $6.6 billion in adults aged ≥60 years, with 44% of the burden due to direct medical costs ($2.9 billion), 18% from indirect costs due to RSV morbidity ($1.2 billion), and 39% from indirect costs due to RSV mortality ($2.5 billion) (Figure 2*B*). Direct medical cost burden per capita was highest in adults aged ≥80 years ($47 300 per 1000 people), while indirect cost burden per capita was highest in adults aged 60–64 years ($66 300 per 1000 people) (Table 2). The trend of higher indirect costs among younger age groups resulted in the total cost burden being the highest in adults aged 60–64 years ($97 800 per 1000 people) followed by ages 65–69 years (79 600 per 1000 people). While only 4% of annual RSV cases were hospitalized, these cases accounted for 94% of the direct medical costs ($2.7 billion). The remaining direct medical cost burden was attributable to RSV cases treated in emergency department and outpatient settings ($179 million). RSV morbidity accounted for 7.9 million days of lost productivity, resulting in $1.1 billion in indirect costs from hospitalized (38% of costs), emergency department (4%), outpatient (27%), and non–medically attended (31%) cases.

### One-way Sensitivity Analyses

**Figure 3.**
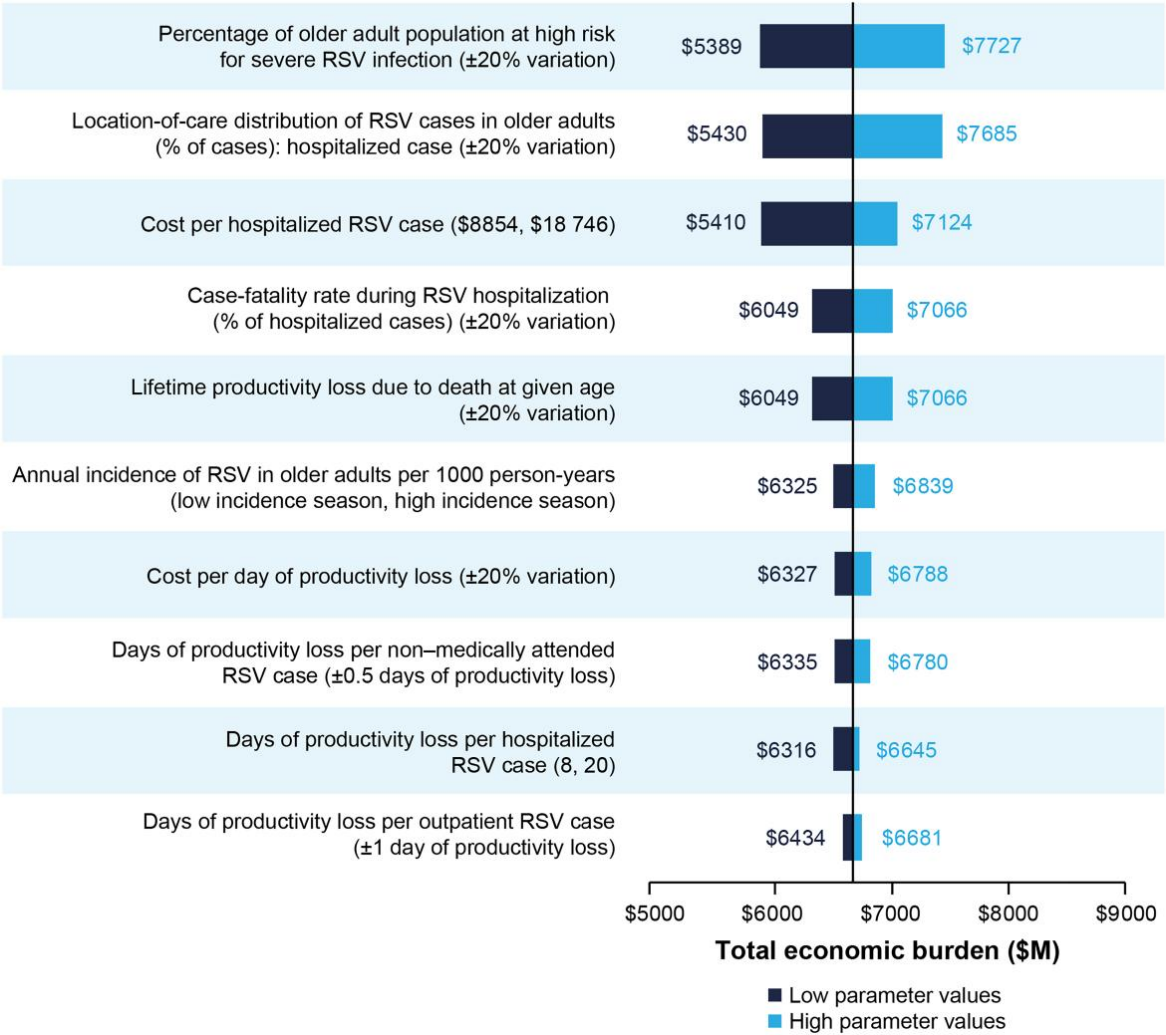
One-way sensitivity analysis results for RSV economic burden in adults aged ≥60 years. The analysis displays model parameters with the largest influence on the annual societal economic burden of RSV. Values in parentheses report the range of values considered in the one-way sensitivity analysis. Abbreviations: ED, emergency department; M, million; RSV, respiratory syncytial virus.

The model parameters with the largest influence on annual societal economic burden of RSV in older adults were percentage of the population at high risk for severe RSV infection, proportion of RSV cases that were hospitalized, direct medical cost per hospitalized case, case-fatality rate during RSV hospitalization, and the lifetime productivity loss due to death at given age (Figure 3).

### Probabilistic Sensitivity Analysis

Probabilistic sensitivity analysis demonstrated significant variability in annual RSV cases (95% UI, 2.7–5.6 million), hospitalized cases (95% UI, 81–320 thousand), and associated annual societal economic burden (95% UI, $3.1–$12.9 billion) because of the collective uncertainty in model inputs.

### Additional Analyses

Among adults aged 50 to 59 years, there were an estimated 20 800 RSV-associated hospitalizations annually, which led to 620 deaths during hospitalization. The total annual economic burden of RSV-associated hospitalization among adults aged 50 to 59 years was $1.1 billion, with 61% of these costs attributable to indirect costs due to RSV mortality ($349 million direct medical costs; $87 million indirect costs due to hospitalized RSV morbidity; $690 million indirect costs due to RSV mortality).

Analyses of health and economic burden from hospitalized RSV cases for individuals aged 18 to 49 years with potential RSV risk factors (Table 3) estimated the number of annual RSV hospitalizations, ranging from 170 for people with CAD to 5620 for people with obesity. Estimated annual total economic burden for those aged 18 to 49 years was the highest for obesity ($358 million), diabetes ($282 million), and asthma ($216 million), which were the potential RSV risk factors with the highest prevalence in this age group (Table 3). However, the annual economic burden per capita was highest among people with CHF ($153 400 per 1000 people), followed by diabetes ($48 000 per 1000 people), COPD ($22 100 per 1000 people), and CAD ($20 100 per 1000 people).

**Table 3.** Additional Analysis Results: Annual Burden of Hospitalized RSV in Adults Aged 18–49 Years With Potential RSV Risk Factors

Outcome	Adults Aged 18–49 y					
	CHF	CAD	COPD	Asthma	Diabetes	Obesity
Total annual RSV hospitalizations	1582	172	332	3394	4423	5618
Total annual deaths during RSV hospitalizations	32	3	7	68	88	112
Total annual economic burden, $M	99	11	21	216	282	358
Direct medical costs, $M	28	3	6	60	78	99
Indirect costs due to RSV morbidity and mortality, $M	71	8	15	156	204	259
Total annual economic burden per 1000 people with potential RSV risk factor, $	153 394	20 122	22 092	9677	47 983	6146

Results cannot be summed across potential risk factors because people may be living with 2 or more of the modeled potential risk factors.

Abbreviations: CAD, coronary artery disease; CHF, congestive heart failure; COPD, chronic obstructive pulmonary disease; M, million; RSV, respiratory syncytial virus.

## DISCUSSION

The current study estimated the annual health economic burden of RSV in US adults. To our knowledge, this is the first study to estimate both direct and indirect costs of RSV in older US adults and the burden from hospitalization due to RSV in adults aged <60 years. Among adults aged ≥60 years, RSV cases were estimated to contribute to an annual $6.6 billion in US economic burden, including $2.9 billion in direct medical costs, $1.1 billion in indirect costs due to losses in productivity from RSV-related morbidity, and $2.5 billion in indirect costs due to RSV mortality. Estimates of economic burden in the current study were most sensitive to risk and costs for RSV hospitalizations, as well as the seasonal variability in RSV incidence due to uncertainty in model inputs.

Annual direct RSV-related medical costs were comparable to those reported by other studies, falling within the range of $1.5 to $3 billion reported by Herring et al among US adults aged ≥60 years [12]. The current study also estimated 1 090 000 medically attended cases, 173 000 hospitalizations, and 8200 RSV attributable deaths, also within the range reported from other studies (0.6–2.3 million medically attended cases, 71 000–238 000 RSV-related hospitalizations, and 4000–43 000 RSV-attributable deaths) [5, 12]. Lower RSV death estimates in the current study relative to Herring et al [12] is likely attributable to methodologic differences, as Herring et al reported RSV-attributable deaths within 12 months of inpatient admission and the current study estimated only mortality during hospitalization.

Results from this study highlight the importance of recognizing the burden of RSV among other respiratory viruses in older adults. Compared with influenza, RSV results in similar or higher rates of severe illness, hospitalization costs, complications, and mortality after hospital admission [1, 24, 33–35]. Influenza accounts for $1.6 to $8.7 billion (2022 US dollars) in direct medical costs in older adults annually, compared with the $2.9 billion in annual direct medical costs from RSV in older adults estimated in this study. Estimated indirect costs have ranged widely for influenza in older adults, ranging from $1.3 to $14 billion (2022 US dollars) due to morbidity and mortality annually [7–10], compared with the annual estimate for RSV in the current study's base-case analysis ($3.7 billion).

Additional analyses were conducted to assess the societal economic and health burden of RSV-associated hospitalization in adults aged 50 to 59 years and adults aged 18 to 49 years with potential underlying RSV risk factors. While estimated hospitalizations were lower in those aged 50 to 59 years (20 750) than estimates for older adults in this study (39 140 in ages 60–64 years, 34 530 in ages 65–69 years, 59 740 in ages 70–79 years, and 39 800 in ages ≥80 years), the $1.1 billion economic burden due to RSV hospitalization in adults aged 50 to 59 years added considerably to the total burden of costs in older adults. Among adults aged 18 to 49 years with potential RSV factors (asthma, CAD, COPD, CHF, diabetes, obesity), the burden per person was the highest in adults with CHF ($153 400 per 1000 people), followed by diabetes ($48 000 per 1000 people) and COPD ($22 100 per person). This substantially higher burden in those with CHF is aligned with studies showing that individuals with CHF are more likely to have longer hospital stays and more likely to be admitted to the intensive care unit due to RSV infection [36]. The risk factors for severe RSV disease have not been comprehensively studied, and identifying groups at risk may assist decision makers to assess which individuals will benefit the most from recently approved RSV vaccines. The estimates from the current study also reveal increased economic burden in older individuals at high risk of severe outcomes from RSV disease, reinforcing the need for safe and effective immunization programs in these subpopulations.

These results must be considered in the context of their methodologic limitations. First, incidence and case severity data were obtained from a landmark prospective surveillance study conducted during the 1999–2003 RSV seasons in Rochester, New York [1]. This reliance on one study is somewhat mitigated by the fact that weighted incidence and hospitalization rates for medically attended RSV cases in the current study were similar to estimates from other studies [3, 37, 38]. It is also likely that estimates in the current study may be an underestimation of true burden because long-term care complications and costs were not fully captured, and limitations in diagnostic testing (eg, sensitivity) may not capture all RSV cases [39, 40]. Additionally, reliance on Medicare data may have led to some cost underestimation, as costs may be higher in commercially insured populations [41]. Future research should focus on the potential cumulative burden of RSV disease in individuals with multiple comorbidities. As people age, the likelihood of having multiple underlying risk factors for RSV increases [20]. Therefore, it is essential to explore the cumulative burden of RSV in this population to gain a better understanding of the impact of RSV. The costs are likely to be underestimated, as persons with immunocompromising conditions were excluded from the analysis, and the CDC also describes this group as high risk for severe disease [22]. Furthermore, as the CDC frequently updates their list of potential risk factors based on new evidence, it is imperative to routinely conduct analyses so that recommendations for interventions against severe disease for various populations can be updated. It should be noted that the additional analyses in adults aged 18 to 59 years estimated the annual economic burden due to hospitalizations only, verses a more comprehensive economic burden estimate, because limited data were available to estimate the burden due to cases treated in other health care settings and non–medically attended cases. Excluding such cases underestimates the total burden of RSV in these populations, particularly for indirect costs, as a previous analysis of the economic burden of influenza estimated that the majority of overall costs in adults aged 18 to 64 years were indirect costs, and the majority of those indirect costs were among nonhospitalized cases [7].

This study suggests that RSV-attributable direct medical costs and productivity losses are significant in older adults aged ≥60 years; additional analyses suggest that hospitalized RSV in adults aged 50 to 59 years and adults aged 18 to 49 years with potential RSV risk factors also add substantially to the economic burden. Recent surges in RSV infections [42] underscore the importance of our results, which may be helpful to policymakers, given upcoming vaccination recommendations for adults. An RSV vaccine would be able to prevent or mitigate the effects of severe disease and its subsequent complications in high-risk populations.

## Notes

***Acknowledgments.*** Data analysis support was provided by Abby Hitchens and Sean Candrilli from RTI Health Solutions. Medical writing and editorial assistance were provided by Meenu Minhas, PhD, of MEDiSTRAVA in accordance with Good Publication Practice (GPP3) guidelines, funded by Moderna, Inc, and under the direction of the authors.

***Author contributions.*** J. C. and K. A. H. made substantial contributions to the conception and design of this work, data collection, analysis and interpretation of data, and drafting and critical revisions of the manuscript. E. W. participated in data analysis and interpretation and writing and critical revisions of the manuscript. C. A. P. contributed to the conception and design of this work and writing and critical revisions of the manuscript. P. G. made substantial contribution to the conception of this work, analysis and interpretation of the data, and writing and critical revisions of the draft. All authors provided final approval of the manuscript.

***Financial support*.** This work was supported by Moderna, Inc.

***Data availability.*** The authors declare that all data generated or analyzed in this study are available within this article.
